# Negotiated Management Strategies for Bovine Tuberculosis: Enhancing Risk Mitigation in Michigan and the UK

**DOI:** 10.3389/fvets.2019.00081

**Published:** 2019-03-26

**Authors:** Ruth A. Little

**Affiliations:** Department of Geography, University of Sheffield, Sheffield, United Kingdom

**Keywords:** bovine tuberculosis, risk mitigation, biosecurity, human dimensions, responsibilisation

## Abstract

Bovine tuberculosis (bTB) is an epidemiologically, politically, and socially complex disease. Across multiple international contexts, policy makers have struggled to balance the competing demands of wildlife and agricultural interests in their efforts to create workable and effective disease management strategies. This paper draws comparative lessons between the cases of Michigan in the USA and the UK to exemplify some of the challenges of developing an effective strategy for the long-term control of endemic disease, particularly reflecting on efforts to “responsibilise” cattle producers and engage them in proactive activities to mitigate transmission risks on their own farms. Using qualitative data derived from 22 stakeholder interviews, it is argued that the management of bTB in Michigan has important lessons for the UK on the role of human dimensions in influencing the direction of disease control. The management of endemic bTB relies on the actions of individuals to minimise risk and, in contrast to the predominantly voluntary approach pursued in the UK, Michigan has shifted the emphasis towards obtaining producer support for wildlife risk mitigation and biosecurity via a mix of regulatory, fiscal, and social interventions. Whilst the scale of the bTB challenge differs between these two contexts, analysis of the different ideological bases for selecting management approaches offers interesting insights on the role of negotiated outcomes in attempts to adaptively manage a disease that is characterised by complexity and uncertainty.

## Introduction

Bovine tuberculosis (bTB) is principally a disease of cattle, but there are several places worldwide where free-ranging wildlife are reservoirs of infection, namely brushtail possums in New Zealand, European badgers in the United Kingdom, wood bison and elk in Canada, African buffalo in South Africa and white-tailed deer in the United States (1). Where the disease has become established, it can have considerable economic consequences for livestock keepers and poses challenges for national governments and agencies in devising a workable and socially acceptable eradication plan. The ultimate rationale for intervention is based on the potential threat *Mycobacterium bovis* poses to public health (2); however, the proximate driver for expenditure on bTB management is the potential economic effect of trade restrictions on milk and meat products (3, 4) and the wider ecological concerns associated with potential disease spread into new regions and ecosystems. The case for eradication has been contested based upon cost benefit criteria and the relative importance of the risk posed to human health [see (5–7)], but it remains the declared goal for many international control programmes [see, for example, (4, 8)].

Experiences from around the world exemplify the challenges faced by disease managers in constructing a coherent, cost-effective, and workable strategy for eradication. Multiple ecological and epidemiological challenges remain [see (9, 10) for a review], but socio-economic and political factors also have a key role to play in influencing the outcomes of disease control strategies; including, the cost-effectiveness of the policies, political will to implement management programmes and the social acceptability of individual control measures. The UK is perhaps the foremost example of the difficulties involved in constructing a control regime under conditions of intense socio-political scrutiny. A primary point of contention has been the decision to cull badgers in England, which are considered to have important cultural associations for the general public [see (11, 12)]. Vigorous debate on the role of badger culling in the control of bTB has resulted in policies that have been considered to lack coherence (13) and a situation where the devolved administrations pursue their own control policies, with differing approaches to addressing the disease in their wildlife populations (14, 15)^1^. This has resulted in what Allen et al. [(10), p. 110] considers this to be part of “the current impasse in bTB control” across Britain and Ireland, with multi-factorial problems inhibiting the national eradication programmes.

Socio-economic and political factors have been highlighted as determinants of success in analyses of international control programmes. For example, Professor Ian Boyd, The Chief Scientific Adviser to the UK government's Department for Environment, Food and Rural Affairs (DEFRA) described bovine tuberculosis as a “sociological problem,” stressing the importance of human dimensions in influencing disease outcomes. Similar claims have been made in review papers on the complexity of bTB control (16) and in studies of eradication attempts in the US (1), Australia (17), and New Zealand (18, 19). These determinants tend to focus on three separate, but interconnected factors: the effectiveness of political decision-making; social acceptability of the policies; and the attitudes and actions of affected stakeholders.

This paper focuses on the experience of bTB control in the US state of Michigan to provide a comparison for current and future policy developments in the UK. Whilst the scale of the problem in Michigan is different to the UK, there are interesting comparators in terms of socio-economic and political factors influencing the perceived success of efforts to achieve effective disease control. For example, Carstensen et al. (1) reported, “public tolerance” and political will were considered to exert significant influence on the control measures available to disease managers in the US. The authors also cite a series of temporal, social, economic, and logistical factors that shaped public and stakeholder attitudes towards aggressive disease control strategies, the limitations that these factors placed on management options and the subsequent implications for bTB eradication from the wildlife reservoirs in the USA. Carstensen et al. (1) concluded that, in comparison to the response to a notable outbreak of bTB in Minnesota in 2006, which successfully prevented the self-sustaining establishment of the disease in wildlife, Michigan has lacked the leadership to initiate more “aggressive” bTB management strategies in both cattle (via, for example, buy-out options for herds in areas of high bTB risk) and wildlife (through substantial reduction in deer numbers via intensive culling).

Without the will to institute more “aggressive” responses to controlling the disease in cattle and wildlife populations, the management of bTB often requires a negotiated management response, based upon the level of funding available and the buy-in from the thousands of individual disease managers (e.g., farmers, hunters, and the like) tasked with controlling the disease over a sustained period. As Miller (20) notes, management of diseases at the livestock–wildlife interface often require long-term engagement using a combination of altered livestock husbandry practices, active disease suppression in wildlife, and prevention of transmission using mitigation techniques. Considerable attention has been given to the development of interventions designed to mitigate the risk of bTB disease transmission between cattle and wildlife [see (21, 22)]. Generally, the research concludes that risk mitigation interventions such as deer exclusion fences have great potential but the challenge lies in farmers modifying their husbandry practices and behaviours (20) including maintaining the integrity of fences and keeping gates closed (23, 24). Risk mitigation measures that rely on stakeholder adoption of preventative behaviours [see (25)], therefore, pose challenges for risk managers in formulating measures that will incentivize positive responses.

Similar issues can be observed in the UK relating to the adoption of preventative biosecurity measures at the farm level. Whilst biosecurity is cited as a key part of the Defra's 25 year Strategy to Achieve Officially Bovine Tuberculosis Free Status for England (2014), multiple challenges remain regarding farmers' adoption of measures to reduce the risk of bTB transmission between cattle and between cattle and wildlife. Farmers can be reticent to implement measures because of the limited evidence surrounding the efficacy of many of the interventions (9, 26, 27); the perceived impracticality of implementing measures on their own farms (28), particularly relating to badger exclusion and isolation of bought in cattle, and the uncertain benefits that will accrue in reducing their risk of a bTB breakdown as opposed to the costs of modifying feed and water sources, installing fences to reduce contacts with neighboring herds or establishing isolation facilities for newly bought in animals. Whilst farmers acknowledge the theoretical importance of biosecurity as a preventative measure, this does not always result in taking action to reduce risks on farm (29–31). Such reluctance to act may be associated with farmers' often-reported “fatalistic” belief that there is little that they can proactively do to prevent a bTB breakdown or that “luck” rather than their own actions has more of an influence on the likelihood of the disease entering their herds (32–34).

Currently, the majority of biosecurity measures outlined in Defra's 25 year Strategy are voluntary, with some additional requirements for farms within badger culling areas and for “persistent” bTB herds. Improving biosecurity on and off farm is stated as an important management goal within Defra's Strategy. As the literature indicates, risk managers will need to formulate measures to address the apparent disjuncture between the acknowledged importance yet under-implementation of risk mitigation measures on farm. Using Michigan as a case study, the objectives of this study were to investigate management approaches, policies and interventions designed to engage farmers in adopting and sustaining preventative bTB biosecurity measures and qualitatively assess their impact in contributing to disease control.

The paper will outline some of the comparative lessons that can be learned from Michigan in their attempts to enhance the on-farm risk mitigation element of their disease management strategies and the policies considered most effective in encouraging proactive disease management at the farm level.

## Methodology

The research focused on stakeholder perspectives on eradication efforts, assessing the relative merits of different policy interventions aimed at disease management and appraising the key factors affecting efforts to achieve bTB eradication. The research approach was based upon 22 in-depth face to face interviews conducted at the end of 2014. Non-probabilistic, purposive sampling [akin to (25, 35)] was used to select interviewees with individuals identified based upon their roles as “experts” and “key stakeholders” involved in the development or implementation of bTB policies in Michigan. This research was part of a wider study that included a further set of interviews in Minnesota; the results of which was not reported here. Interviewees were stratified into the following three broad categories: agency professionals involved in bTB management in cattle or wildlife (wildlife managers, programme coordinators, field veterinarians, and communications specialists); university academic and extension personnel; and cattle producer and wildlife stakeholders involved in implementing management practices on the ground. Interviews were conducted in the State capital and in the Modified Accredited Zone (MAZ) in the northeastern lower peninsula (NELP) of Michigan, concentrating on the counties of Alcona, Alpena, Montmorency, and Oscoda Counties.

The research was designed to be a qualitative, in-depth assessment of bTB management approaches in Michigan. As indicated by Naylor et al. [(36), p. 286] “interviewing is the method most often adopted to explore potentially sensitive and controversial issues… and are often commended as a research method for their flexibility and ability to explore difficult issues in a comprehensive and sensitive manner.” Unlike the standardised and structured approaches of farmer attitude surveys or Q-Methodology [e.g., (35, 37, 38)] the interviews were semi-structured and discussions were based around a set of themes within an interview guide; this approach has been used in equivalent qualitative studies on bTB and biosecurity [see (32)]. The interview guide consisted of questions relating to the participant's role in bTB control; overview of the factors influencing the relative success of bTB control (including identifying effective policies and interventions); identification of key stakeholders and their positive or negative contribution to disease management; modes of risk communication and the challenges and successes encountered in promoting “best practice” in disease mitigation; and lessons learnt from their experience of managing bTB in Michigan^2^. Each interview was tailored to the expertise and knowledge of the interviewee and so the focus of each discussion was context specific. However, all interviewees were asked about and responded to questions on policies and interventions that were considered to be effective in encouraging disease managers (e.g., farmers and hunters) to adopt positive disease management practices. The results of which are reported here.

Interviews were digitally recorded (with the participants' informed consent) and later fully transcribed. The data was manually coded in order to develop an empirically grounded coding framework, guided by the key research questions. This involved an iterative and in-depth process of “careful reading and re-reading of the data” [(39), p. 258], beginning with an informal reading of the materials to identify an initial set of high-level thematic codes. The approach followed the conventions of Seidel and Kelle (40) quoted in Basit [(41), p. 144] who “view the role of coding as noticing relevant phenomena; collecting examples of those phenomena; and analyzing those phenomena in order to find commonalities, differences, patterns and structures.” Categories were developed via a process “data distillation” (42) to organise the coded data into meaningful overarching themes. The themes were based upon concepts from existing literature and from words and phrases used by the interviewees e.g., notions of responsibility and responsibilisation; social networks and peer example; drivers and incentives. These themes are represented as organising concepts in the results section.

Following a broad introduction to bTB management approaches in Michigan, an overview will be provided of the Wildlife Risk Mitigation project, which was identified as being a key development in efforts to enhance on-farm biosecurity activities.

## Results

### Management Approaches for bTB in Michigan

On-farm Wildlife Risk Mitigation (WRM) is part of a wider approach to bTB management in Michigan, including surveillance, and control measures aimed at reducing the disease burden in both cattle and wildlife (white-tailed deer). The focus of this paper is WRM, however a brief overview of the control programme is described here.

**Figure 1:**
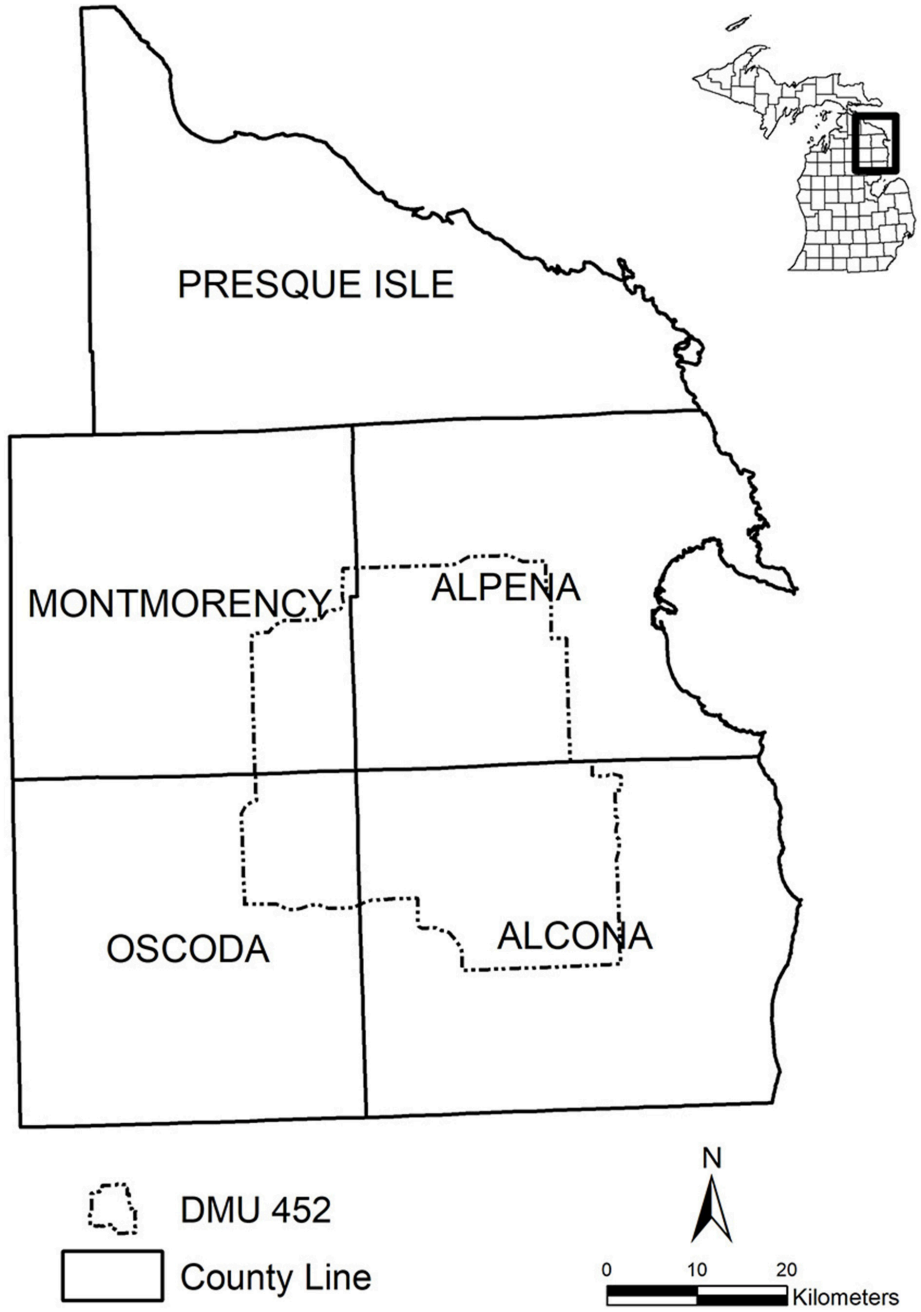
Deer Management Unit (DMU) 452, in northeastern Lower Peninsula of Michigan, USA.

Michigan was declared free of bTB in cattle and bison in 1979. However, in 1975, and again in 1994, bTB was identified in one wild white-tail deer in the NELP of Michigan. Subsequent testing revealed the disease to be endemic in the white-tail deer population within five of the most north easterly counties of the Lower Peninsula. Since 1995 surveillance and testing has been carried out in the affected area via annual surveillance of hunter harvested deer. To date, the disease has been confirmed in nearly 875 of over 254,000 free-ranging deer tested in Michigan, with 77% of bTB-positive deer found in a core area—Deer Management Unit 452—in the NELP of Michigan, where the counties of Alcona, Alpena, Montmorency, and Oscoda meet (Figure 1). Reduction in deer density within the affected area is a key part of the policy, with enhanced measures introduced over successive seasons designed to maximise legal opportunities for the public to harvest deer. These strategies include liberalised hunting seasons; issuing landowners Deer Management Assistance permits to supplement hunting licences; providing disease control permits to cattle producers and non-agricultural landowners in high prevalence areas; and, most recently, the introduction of the Hunter Access Program, to match hunters in search of places to hunt with agricultural landowners seeking additional deer harvest on their land. Deer baiting and feeding bans are also in operation in some of the affected areas.

Following the identification of bTB positive deer in the 1990s, the reinstatement of cattle testing in the affected area revealed the first infected cattle herd in June 1998. Michigan subsequently lost its bTB free status in June 2000 and state-wide surveillance testing was instituted from 2000 to 2003. The Upper Peninsula regained bTB Free status in 2005 and 57 counties in the Lower Peninsula regained bTB Free status in 2011. Surveillance testing identified a core disease outbreak area in 11 counties in the northeastern tip of the Lower Peninsula; since October 2014, seven more of those counties have been declared bTB Free for cattle, leaving 4 remaining. Annual testing of all livestock (cattle, goats, bison) and captive cervids remains in place in the 4 counties (classified as the MAZ by the US Department of Agriculture's Animal and Plant Health Inspection Service-Veterinary Services Branch^3^), with risk-based testing applied throughout the remainder of the State. In the MAZ, the traceability and movement of livestock is regulated through movement permits obtained from the field offices of the Michigan Department of Agriculture and Rural Development (MDARD), electronic identification of animals and annual herd inventories to reconcile discrepancies between animals on farm and official records. Other policies governing livestock movements and limiting deer-cattle contacts will be covered more fully in the following section.

### Wildlife Risk Mitigation

WRM is now a key element of the bTB management strategy, particularly concentrating on the commercial farms in and around the MAZ in the NELP of Michigan, identified as at risk for bTB transmission from wildlife. The policy began as a series of small scale activities at Michigan State University (MSU) which, from 2008, formalised into a voluntary initiative developed by MDARD, MSU Extension, United States Department of Agriculture (Veterinary Services, Wildlife Services), and the Natural Resources Conservation Service (NRCS), with some input from industry. The objective of the programme was to assist producers in identifying high risk areas and practices on their holding and develop plans to reduce the risk of cattle-wildlife interactions. The approach was designed to form part of the “safety nets” (44) put in place to control the disease, complementing the surveillance testing and movement restrictions in helping to prevent opportunities for infection; the ultimate aim being to draw down the disease incidence in cattle.

The programme required changes to be made to management practices and farm infrastructure in the endemic area. It relied upon the development of a series of interventions to assist and influence the implementation of risk reduction measures on farm, including the introduction of hoop barns and deer-proof fencing to protect stored feeds and actions related to cattle accessing feed and water sources. The changes required at farm-level meant that the concept of WRM was controversial from the outset. According to a Michigan policy lead, “*this was probably the most controversial thing that happened in the course of the bTB programme; more so even than testing…there was a tremendous amount of angst and anger about this wildlife risk project.”* Producer concerns focused on the practicalities of excluding deer from their property; the cost of implementing the measures and a perceived inadequacy on the part of Department of Natural Resources (DNR) to deal effectively with the disease in wildlife (e.g., through the reduction of deer numbers). Due to the contentious nature of the proposal, the policy making process involved a series of meetings to develop proposals and standards which were an acceptable compromise between what was desired by policy makers and risk managers and what was considered achievable in practice by the agricultural industry. The process was described in the following terms by an individual involved in the development of the scheme:

“*it's that idea of, okay, if you can't build 20 foot or 12 foot high barbed wire fences all the way round …where are the opportunities to reduce risk most cost effectively? So we got the best available science from [Michigan's bTB Programme] and we started sharing it with our stakeholders, the producers and let them decide.”*

The process of negotiation, over a series of three meetings, focused on achieving a balance between an epidemiological ideal and an implementable policy. The process was facilitated by MSU staff as intermediaries and the University published the document.

### Implementation

The implementation of the scheme was described by its instigators in terms of a phased approach, based upon the principles of adaptive management [see (45–47)]: phase one was aimed at individuals identified as “early adopters” who were engaged with a prototype version of the WRM intervention; the second phase was an expansion of the programme, designed to appeal to “capable learners”; and the third was regulatory enforcements to draw in those who were “resistant to change.” It was also phased in regionally; MDARD concentrated on the outlying areas first, where there was an opportunity to elevate the accreditation status more swiftly (for example, in Michigan's Northwest Region where bTB was not endemic in wild deer) and moved on to the more challenging and higher risk area of the MAZ over time. This incremental approach evolved into an increasingly statutory regime and relied on a number of key push and pull factors designed to maximise participation in the scheme. A combination of one-to-one assistance, co-funding of risk mitigation measures (such as deer fencing) and restrictions placed on market access have been employed to both encourage and enable producer engagement in the scheme, but also to make it challenging for them to stay outside of the system.

The WRM project is designed around a five-step process which aims to bring livestock producers and technical experts together to create a tailored on-farm plan to reduce the risk of infection between cattle and wildlife. Producers are offered an educational meeting before completing an on-farm risk assessment. The risk assessment is conducted between government agency staff and is designed to be both educational (recognising potentially risky areas, and practices on farm) and regulatory, with the implementation of certain mitigation actions being classified as compulsory. Once the WRM Action Plan has been agreed, the producer then indicates a timescale within which they propose to complete the actions. Depending on the risks identified on farm, these actions may include interventions to limit potential infection transfer at sites where cattle are fed (governing where, how often and how much cattle are fed), water sources for cattle and where cattle feed is stored. Each of these sites have been identified as a risk for disease transmission (48, 49) and so require changes to management practices, including fencing off feed and water sources to prevent deer access. Once the plan has been implemented, the work is subject to an annual verification process to check that the interventions and actions are still in place.

As part of the development of the plan, a cost-sharing scheme was introduced to assist cattle producers in implementing the actions. During 2008–2013, over $3.6 m was expended on WRM measures. Government, state and federal funds accounted for $2,637,000 of this figure and a further $1,002,000 was contributed by cattle producers. In the early phases of the scheme, 50% of the cost-share funding came from the state and the USDA, and in the later phases, the bTB programme utilised the USDA, NRCS's Environmental Quality Incentives Programme. The benefit of the latter approach being that mutual aims could be achieved from a single funding allocation and that the conservation office, which already had close historical links to the farming community, could take over the responsibilities for the continuation and annual verification of the scheme.

### Drivers and Incentives

The development of the risk assessment and verification process was originally badged as a voluntary approach. However, (dis)incentives were introduced to influence the level of uptake amongst producers. One interviewee described it as, “*incentives on the cattle side were, first of all, it was disincentives, you couldn't move [cattle] if you didn't do it*.” Additional testing and restrictions on market access were the primary levers to encourage uptake of the WRM. The policy stipulated that a pre-movement test be carried out on cattle from non-WRM farms, with a further post-movement test 60 to 120 days after purchase being required of the purchaser at their own expense. The rationale for the approach was described by an individual involved in developing the policy as follows:

“*So the state used to pay for all that [testing] and in these counties we've said okay, you know, you have an hour, you could get a biosecurity plan and you don't have to do this test, but, you know, if you don't want to do that that's fine, you can do this additional test, but you get to pay for it now and then the guy who buys your cows, unless he gets them slaughtered, has to also do a test at his expense. Well that means that the cattle are discounted, because when people go, oh, I got to do a test, well that's going to cost me something, so I'm not going to pay quite so much for these cattle and so that has driven some people and we were trying to use market forces to, you know, move people towards doing the right thing.”*

Through restricting market access and attaching a financial disincentive to the cattle from non-WRM farms, the aim was to shift producers' assessment of the costs and benefits in favour of enrolling in the WRM scheme. The (dis)incentives were strengthened in January 2015, when regulations were introduced stating that all farms in the bTB core area must be wildlife risk mitigated; otherwise, these producers could only send their animals to slaughter.

### Social Networks and Peer Example

In addition to perceived economic (dis)benefits, risk managers involved in developing, and refining Michigan's eradication programme employed a series of techniques to influence the social context into which their strategies were being placed. The approach included the use of existing social networks within the locality to promote sign up to the scheme and peer example coupled with “teachable moments” to encourage producer-advocates of the scheme to explain the benefits, particularly following cases of bTB outbreaks where WRM may have been assistive in preventing disease transmission. The rationale being, as summarised by an extension agent, “*peer example, call it, rather than peer pressure, can be very effective.”* The use of social networks was seen as a way of dealing with the negative view towards government officials and enlisting more trusted intermediaries to deliver the message on the benefits of the scheme. This approach is exemplified in the following quotes:

“*I think other people have said okay yeah if I'm hearing this from my neighbour and my friend I'm not hearing it from the state veterinarian or, you know, some USDA regulator, but I'm hearing it from, you know, my friends and they tend to take it a little bit more seriously, especially if you're seeing that person every day or at church or in a grocery store or at the bar or whatever, so that makes it a little bit more real”*.

“*So one of the things we did, we had I don't know about maybe 45, 46 of these that were still hanging out here in the farms in here that had not done a biosecurity plan and so back in April I made phone calls to people that work on these farms and just trying to ascertain who is the person that might most effectively communicate things in a positive way, where we would get them actually to do something and so actually some of our guys, you know, are relatives to these people or they've cultivated, you know, decent relationships.”*

The role of these gate keepers within the producer community was important to facilitate wider implementation, using existing social networks to connect government authorities with producers at the farm level. There were also particular individuals that were highly functional in terms of engaging producers and hunters in disease management efforts, be they as an identifiable, visible, and approachable lead of the bTB programme or as key personnel within the areas most at risk from a bTB outbreak. In the words of one policy maker, “*[t]he policies were supporting the risk mitigation, the policies were making sure you had some local expertise, it wasn't just coming out of Lansing to talk to people.”* The division of “distant” government officials in the State Capital of Lansing and the affected communities in the NELP was addressed through convening local meetings, placing the onus on appointing personnel from within the local area and working through MSU extension, which has long-established links with cattle producers via existing research programmes and community outreach.

### Sustaining Disease Management Practices

During the development phases, it was recognised that the installation of measures such as deer fencing was only the first part of a successful WRM plan. The second part was the maintenance and continued use of measures by cattle producers, such as keeping gates to feed sources closed. The challenge of sustaining disease management practices at the farm level was described in the following terms:

“*How do we get producers to do that, how do we support it, you know, how do we maintain it, because, you know, you can pour a lot of money into fencing and, you know, other mitigation, but if you do it for 1 year and then you say it's too much trouble, you know, to keep the fences maintained and stuff like it doesn't really matter then, so it's not only doing the mitigation, but then maintaining it over time.”*

To address this challenge, conditions were attached to the grants allocated for co-funding of WRM measures. Producers were required to sign a contract outlining their obligations (e.g., closing gates) and if they were found to be in contravention of those conditions, then the state would be entitled to reclaim the cost-share money and the farm's WRM verification would be withdrawn, with consequent implications for trade and enhanced testing.

### Promoting Action and Assessing Impact

WRM began as a controversial policy aimed at enhanced risk mitigation at the farm level. As already noted, the development was controversial because of the implications that the new measures and requirements had for farm management decisions and infrastructure. During interviews, stakeholders reflected on the difficulties involved in introducing and implementing the scheme, but also recognised the perceived benefits that WRM provided in terms of enhanced disease management through reducing risk at the livestock-wildlife interface and the transfer of responsibilities for disease management to producers on their own properties. The following section provides an overview of stakeholder perspectives on the perceived utility and impact of the WRM scheme.

### Responsibility

A clear reason for the development of the WRM scheme was to re-centre the responsibility for keeping bTB out of herds back into the hands of the cattle producers. Whilst WRM has been a predominantly government-led scheme (with input from producers and producer organisations), the aim has been to highlight what producers can do on their own holdings to mitigate risk and then, via co-funding and advisory visits, enable them to implement exclusion measures such as barns and deer fences. This represented a step change in the policy. In the words of a field veterinarian:

“*I mean before [WRM] it was just test, test, test, test, test, test, find it, where do we find it? And it wasn't until the wildlife risk programme started that we started having something to say hey, let's do something to help prevent it”*.

The emphasis on engaging producers in proactive action was driven by a number of considerations: first, the need for producers in the NELP to act in the interest of the rest of the cattle industry in the state of Michigan (to retain interstate market access); and second, the realisation that deer would remain only a partially controllable element of disease transmission due to a perceived—on the part of the cattle industry—lack of social and political will to reduce deer densities. Producers were, therefore, encouraged to look at what they could do on their own holdings to institute some control over the opportunities for transmission within the farm boundaries.

Whilst the aim was to transfer responsibility for mitigating risk to individual producers, the initiative remained government-led. Through the implementation of market-driven interventions, co-funding opportunities and increasingly statutory measures, the onus for compliance came from a regulatory source. Thus, replacing the previous approach of leaving it to individual farmers to assess and institute risk management on farm and relying on peer pressure amongst producers to encourage uptake. When asked about the role of peer pressure, a cattle producer commented:

“*It's not so much peer pressure as it is pressure on the government or those above to make the policies that'll force them into it, yeah, that's more the pressure than me going over. I don't want to go over to my neighbour and tell him you have to do this, you know, I can go over there and nicely tell him why he should do it, but for me to go tell him he has to do it I don't want to do that, I don't want to put myself in that spot either.”*

Engendering greater responsibility for assessing what was possible on individual holdings and underlining producers' ability to exert some control over their own situations was an important driver. This was, however, coupled with a more top-down approach of imposing market and regulatory conditions to promote and embed management changes across areas most at risk from a bTB breakdown.

### Assessing the Impacts

WRM was designed as a management strategy to reduce rather than eliminate risk on farm, placing the emphasis on taking greater control over limiting opportunities for deer-cattle interactions and working with producers to focus on the elements within their control to promote effective management of deer-cattle interactions. In terms of benefits, interviewees cited a greater awareness amongst producers of the risks posed to their own farms and enhanced actions around careful storage of cattle feed, with wider general improvements to biosecurity. Whilst being unable to provide evidence for or quantify the benefits of WRM, an assumption was shared amongst interviewees that decreasing the risk of contacts would decrease the number of cases. This opinion is exemplified in the following quotes—the first from a member of the USDA's epidemiological research team and the second from a cattle producer in the high risk area of the MAZ:

“*Well if the producers are compliant with their plan it has I believe reduced the wildlife livestock interface quite a bit and it's also made people I think more aware of how the disease transmission could occur and what they need to do to decrease the amount of contact that the cattle have with deer.”*

“*Well the risk mitigation I believe has worked. It's not foolproof, but it has helped. If nothing else has brought it to the people's attention that these are the focus areas that they should focus on, you know, keeping the feed away and that type of thing. It's brought some attention at least that way and I think some people are becoming more receptive to “agriculture's going to have to take some role in this.” I mean when this first started Ag kind of stepped back and said this is their [the DNR's] problem; let them deal with it and it'll work out when they work out their problem. Well obviously, we're not going to reach that point, so we have to step up to the plate and do our part too. Now we have different opinions on what our part is, you know, every person has a different opinion what they're willing to do and capable of doing.”*

Both of these quotes raise the issue of producers' implementation of the stipulated measures, and is indicative of a wider theme of discussion on compliance with the control regime. Producers and those involved in the preparation and verification of individual farm plans, stated that WRM tended to be based upon a negotiation between the ideals envisaged by state agencies and the practicalities of what was considered achievable at the farm level. This process was described by producers as a form of “trading” back and forth to find a plan that was acceptable to both parties. Finding this middle ground for WRM was considered to be more constructive than imposing a set of measures that were deemed unattainable by the producer and which may prompt non-compliance. As one producer commented,

*I'm sure [MDARD] would like us to tighten up a lot of our standards… but then nobody's going to follow through with it…. our standard might not be exactly as high as we want it to be, but if it'll address 50% of the risk and they'll do it 100% of the time; that's better than addressing 90% of the risk and doing it none of the time*.

The same producer stated that, if measures were too onerous, there would be a temptation to make sure that the farm seemed compliant for the winter inspection, but that the effort would not be sustained throughout the remainder of the year.

In addition to reporting that the prevailing opinion had become one of grudging acceptance within the industry, the interviewed producers also raised concerns about what they considered to be the negative consequences of WRM. Issues cited included the reduced carrying capacity of farms (due to restrictions on grazing and availability of land for harvesting winter forage in areas considered attractive to and frequented by wild deer) and the negative implications for smaller producers who were less able to absorb the costs of complying with the new management regime. Whilst lower stocking densities and removing smaller producers less able to comply with WRM regulations may have positive benefits for the programme as a whole, the social implications of “it hurts some people” was raised as an issue.

A final point of note was the importance of risk perception in sustaining the momentum of the programme. The perception being that, as the sense of risk associated with tackling bTB decreases, the levels of complacency in sustaining disease management efforts increases. The risk of complacency was considered a high priority when developing a control strategy for a disease where endemic infection in the wildlife population persists. Progress towards eradication ultimately depends on a long-term commitment from multiple stakeholders (including producers, hunters, state agencies, and the federal government) to implement mitigation measures, provide adequate economic and political support for sustained management interventions and sustain the policy direction towards a goal that may take decades to achieve.

## Discussion

This paper has highlighted that bTB is an epidemiologically, socially and politically complex disease, creating multiple challenges for disease managers in constructing a coherent, cost-effective and workable strategy for eradication. This complexity is particularly pertinent in countries where the disease has become endemic in cattle and wildlife populations, demanding a long-term, multifactorial approach that is dependent upon a comprehensive set of control measures, sustained political will, adequate funding, stakeholder involvement and acceptance of interventions. Michigan and the UK have been highlighted as examples of how this complexity has played out in practice and underlines the case that the development of bTB management strategies need to be viewed as a social as well as scientific undertaking. This argument is in line with the analysis of Gormley and Corner (50) who point to the key role of stakeholders in bTB eradication programmes around the world and underlines calls for interdisciplinary research [e.g., (51–53)] and the development of viable management solutions based upon socio-technical approaches and interventions.

### Enhancing Engagement

Human dimensions have been recognised as a key factor influencing the relative success of management approaches (17, 19, 54) with research efforts focusing on the role of public acceptability of wildlife control measures, the attitudes and actions of stakeholders (38, 55, 56) and the adoption of preventative biosecurity measures at the individual farm level. A central research theme, particularly in the UK, has focused on the adoption of biosecurity interventions and efforts to enhance opportunities to limit disease transmission between cattle and between cattle and wildlife at the farm level. Research has highlighted key reasons for the under-implementation of measures, including fatalism, uncertainty and scepticism on the practicality and efficacy of biosecurity interventions and, consequently, an unclear cost-benefit analysis of spend vs. gain. Critically, in an endemic disease situation, progress towards eradication will depend upon sustaining risk mitigation efforts over long periods, depending on the cooperation and buy in of producers and key stakeholders. The research reported here sought to provide an analysis of how risk mitigation became embedded within the state of Michigan's eradication programme and uses stakeholder narratives to identify key components that were considered effective in generating change.

The literature review identified a specific challenge for risk managers: formulating measures that incentivise positive and proactive risk management actions from stakeholders (25). The findings presented here identified Michigan's WRM programme as a step change in the state's approach to disease control. Interviewees identified the programme as a means to transfer some of the responsibility to producers to take a more proactive approach towards risk mitigation, first relying on voluntary uptake and then moving to more statutory measures. Social as well as technical processes were developed to address some of the barriers to change identified in the social scientific literature. For example, WRM was used as a tool to shift the uncertain cost-benefit of instituting biosecurity measures through introducing market and regulatory (dis)incentives; “trusted intermediaries” were identified to communicate with producers, recognising the lack of trust and confidence in government agencies to eradicate the disease (57–60) and finally, questions of practicality and efficacy were addressed by working with individual producers to highlight opportunities for change, facilitating their implementation via co-funding and enforcing change where necessary. WRM is essentially a government-led programme with regulatory backing, but the creation of individual farm plans is based upon a negotiation, balancing the epidemiological ideals of risk mitigation with the willingness and ability of producers to institute what are considered to be practical and acceptable interventions on their holdings. Interviewees could not provide evidence of the effectiveness of WRM, but considered it to be successful in changing the management approach towards more actively involving producers in the control strategy for mitigating their own risks.

When drawing comparisons between Michigan and countries with areas affected by endemic bTB such as the UK, there are limitations that should be recognised when offering any “lessons learnt.” First, this is a relatively small qualitative study which was designed to be illustrative rather than representative of stakeholder views. Second, the scale of Michigan's bTB problem is very different to that of the UK, with only 5–6 cases per year in the cattle herd and a prevalence of around 2% in the deer population (47). For example, in 2016, 4 beef herds, 1 feedlot, and 1 dairy herd within the MAZ were found to be bTB positive, which was considered a “spike” in incidence of infected herds (54). By comparison, in the same year, there were 3,753 new bTB incidents in England alone (61). Third, as with any international comparison, there is a difference in the political context for decision-making; particularly relevant in this case is the need for the state of Michigan to conform to Federal requirements established by the USDA, which govern the acceptable level of bTB prevalence and is the ultimate arbiter for restricting or enabling interstate trade of cattle. The different pressures applied and the balance established between maintaining a viable cattle industry and eradicating bTB are important contextual factors in guiding the policies pursued in charting a course towards eradication.

Whilst recognising these caveats of generalisability, scale and differing political contexts, the Michigan experience does offer an interesting case study in negotiating the challenges of shifting the focus beyond testing and surveillance towards obtaining producer engagement in WRM and farm biosecurity. Defra's Strategy to Achieve Officially TB Free Status for England similarly recognises the need to engage farmers in reducing their risk through careful cattle purchasing and limiting opportunities for transmission between cattle and between cattle and wildlife. However, the Strategy largely remains split between the application of statutory control measures—including continuous surveillance of cattle herds, removal of bTB test reactors and other cattle suspected of being infected with bTB and movement restrictions for bTB breakdown herds—and a predominantly non-statutory (voluntary) approach towards biosecurity implementation. In recognition of the persistent challenges surrounding biosecurity implementation [see (31)], there are ongoing discussions to identify mechanisms to encourage herd owners to take additional steps to improve their purchasing and biosecurity practices, including linking compensation to membership of herd health schemes such as the Cattle Health Certification Standards (CHeCS) scheme (62) and investigating means to give “earned recognition” to farmers for verifiable good biosecurity practices [see (63–65) for context]. This represents a movement towards rethinking the governance of biosecurity, but remains dependent upon the voluntary enrolment of farmers which, to date, has resulted in limited sign-up to the Bovine TB Herd Accreditation element of the CHeCS cattle health scheme. Clearly, as was the case in Michigan prior to the introduction of WRM, the challenge of achieving sustained farmer engagement remains unresolved and potentially requires a rethink of the socio-technical mechanisms by which this could be achieved.

### Responsibilisation

Developing a greater sense of responsibility for biosecurity management is an important theme in both the Michigan case study and in policy narratives in the UK. As reported in the work of multiple social scientists, the “responsibilisation” of a wide range of actors beyond government is a process closely linked to the increasing neoliberalisation of animal health management, shifting the onus on to industry and farmers to manage their own risks through enhanced “biosecure citizenship” (66–69). This reflects wider trends in international policy development towards “empowering” citizens to take greater control of their own individual and community well-being in, for example, making themselves less vulnerable to crime through changing their actions and routines to minimise their potential exposure to risk, or making proactive changes to diet and exercise to mitigate future health risks (70, 71).

Whilst the principle of enhanced responsibility is a common theme between the Michigan and UK policy landscapes, the mechanisms to achieve change are different. As Enticott et al. (27) report, the UK model of promoting biosecurity has developed within a political context based upon an ideological reluctance to regulate and has increasingly relied upon theories of behaviour change designed to “nudge” farmers towards taking action via the use of social norms and provision of information to guide choices [see also (72, 73)]. Examples include the introduction of ibTB—a publically available web-based interactive map showing the locations of bTB breakdowns and breakdowns resolved in the last 5 years, in England [see (74)]—and the promotion of the principles of risk-based trading to encourage farmers to make “informed” cattle purchasing decisions and reduce the risk of introducing disease via trade (75–77). This strategy is essentially voluntary, based upon improved communications to heighten awareness towards mitigating risks and operates as a “population strategy” [see (27)] using universal biosecurity principles to convey what should be “best practice” rather than considering applications that are more specific to individual farm contexts. Conversely, Michigan has moved towards a mix of regulatory, fiscal and social interventions that attempt to fit the ideals of standardised biosecurity protocols to specific farm contexts on a one-to-one basis (54).

The neoliberal logic of devolving biosecurity governance to industry and individual farmers has been questioned in the social scientific literature, citing farm-level and institutional factors as reasons why enhanced participation is unlikely to occur [see (78)]. For example, the approach assumes that farmers are willing to take on the additional responsibility and associated actions and that they have the knowledge and resources to implement the changes on their own holdings (ibid). Research suggests that this is not the case, as stated concerns for better biosecurity are not being translated into practice [e.g., (28, 31, 35)]. The reasons cited in Higgins et al. (78) include: farmers considering their biosecurity to already be of a satisfactory standard; concerns over the evidence base underpinning biosecurity interventions and the perceived controllability of the disease [see also (79)]; the applicability of universal biosecurity recommendations to individual farms; and the opinion that biosecurity is essentially a “government issue” with suggested biosecurity actions representing an external solution to an externally imposed problem. Taking each of these issues into account, and adding the unclear cost-benefit of biosecurity applications for bTB, there is a clear lack of incentives for taking voluntary action, often leading to uneven application of measures; the result of which is currently an unknown in terms of its effect on the UK bTB disease control regime.

### Incentivising and Sustaining Change

The Michigan case study responds to a number of these critiques through creating a clearer rationale for incentivising changes to biosecurity practices. It also answers concerns about the utility of a one-size fits all set of recommendations that runs counter to farmers' view that these measures are impractical to implement and that they do not solve the complexity and uncertainty that are inherently linked to the disease. In a study of the Biosecurity Intensive Treatment Area (ITA), developed by the Welsh Assembly Government in 2006, Enticott et al. (27) highlighted the limitations of universal biosecurity practices and the difficulties of inspiring behavioural change with broad-scale knowledge. Instead, the authors advocated for an approach that matches solutions to individual farms via a more discursive process between farmers and advisors. Much like the conclusions reached in the case study presented here, Enticott et al. [(27), p. 334] state that “whilst some biosecurity interventions may make veterinary sense, without the support of the farmer and the wider social environment there is little point suggesting them for they will be rejected.”

Incorporating processes of discussion, negotiation and accommodation to individual farm contexts may introduce concerns about diluting potential management outcomes. However, as Enticott (26) and Higgins et al. (69) suggest, finding a balance between standardisation and negotiation may provide options for progressive and responsive solutions that incorporates the challenging component of social complexity into management responses. As multiple authors and policy makers have stated, people and their actions are critically important factors in influencing the trajectory of bTB control and progress towards eradication. Using existing social scientific evidence on the institutional and farm-level factors that both promote and undermine efforts to enhance biosecurity responses should be the first step in devising, implementing, and evaluating different approaches towards embedding interventions that are capable of creating and sustaining proactive management options for bTB.

## Conclusion

The aim of this paper was to draw comparative lessons between the cases of Michigan and the UK to exemplify some of the challenges of developing an effective strategy for the long-term control of endemic disease, particularly reflecting on efforts to “responsibilise” cattle producers and engage them in proactive activities to mitigate transmission risks on their own farms. The study was designed to respond to prominent themes in the social scientific literature that identified a range of socio-political and economic factors inhibiting the implementation of risk mitigation measures on farm; an issue that is particularly critical in areas with endemic bTB. The results indicate that in contrast to the predominantly voluntary approach pursued in the UK, Michigan has shifted the emphasis towards obtaining producer support for wildlife risk mitigation and biosecurity via a mix of regulatory, fiscal, and social interventions. Whilst there is a common goal of transferring responsibility to producers to exert control over their own transmission risks, Michigan's WRM exemplifies a socio-technical approach that goes beyond highlighting what producers can do (through information and communications campaigns) to incentivising and promoting change via market (dis)incentives, co-funding, utilising social networks and tailoring approaches to individual farm contexts.

Neoliberal approaches designed to “responsibilise” cattle producers have been identified as problematic because the approach assumes that farmers are willing to take on the additional responsibility and associated actions and that they have the knowledge and resources to implement the changes on their own holdings. Taking these issues into account, and adding the unclear cost-benefit of biosecurity interventions for bTB, there is arguably a need to create a clearer rationale for incentivising changes to biosecurity practices in the UK. Whilst the scale of the bTB challenge differs between these two contexts, the development of WRM in Michigan offers instructive lessons in creating a clearer rationale for incentivising changes to biosecurity practices and offers interesting insights on the role of negotiated outcomes in attempts to adaptively manage a disease that is characterised by complexity and uncertainty.

## Ethics Statement

This study was carried out in accordance with the recommendations of the University of Sheffield ethical review panel with written informed consent from all subjects. All subjects gave written informed consent in accordance with the Declaration of Helsinki. The protocol was approved by the University of Sheffield ethics committee.

## Author Contributions

RL collected the data, came up with the concept for the manuscript and drafted the content.

### Conflict of Interest Statement

The author declares that the research was conducted in the absence of any commercial or financial relationships that could be construed as a potential conflict of interest.

## Footnotes

^1^Animal health is a devolved issue in the United Kingdom. England, Wales, Scotland, and Northern Ireland each have the ability to develop and implement their own control policy for bovine tuberculosis, which is currently subject to oversight and audit by the Food and Veterinary Office of the European Commission. It should be noted that Scotland has been Officially Tuberculosis Free (OTF) since September 2009.

^2^For study replication purposes, the interview guide is included as a Supplementary Data File. Full details of the sampling, research approach, and anonymized transcripts can be found within the ReShare UK Data Service repository.

^3^The prevalence of infection a State or zone are classified in five categories: (1) Accredited-free state or zone; (2) Modified Accredited Advanced state or zone; (3) Modified Accredited state or zone; (4) Accreditation Preparatory state or zone; and (5) Non-Accredited state or zone [see (43) for an explanation of the United States bTB accreditation categories].
