# Supplementary material for: Negotiated Management Strategies for Bovine Tuberculosis: Enhancing Risk Mitigation in Michigan and the UK
Source: Front Vet Sci. 2019 Mar 26;6:81. doi: 10.3389/fvets.2019.00081 (PMC6443979; doi:10.3389/fvets.2019.00081)
Supplement: Supplementary file 1 [file Data_Sheet_1.PDF]

Interview Schedule

***Introductions** – Aim of the wider project and objectives of the US fieldwork. Talk through the ethical approval form and note that they can withdraw at any time. Ask the participant to sign the form.*

1. Participant's role in TB control – approx 5 minutes

Can you please give a brief description of your role with regards to TB control?

- Length of time in the position
- Geographical scope of their role

2. General overview of TB control and the factors affecting disease control – approx 20 minutes

How successfully is TB being controlled in your state/county/locality?

What has contributed to the success?

What has limited the progress towards controlling TB?

Looking in more detail, what are the factors that affect the adoption of disease mitigation measures at different scales of authority?

- Federal level
- State level
- County level
- Farm level
- Other

Can you point to any examples of where a **policy** or the introduction of certain measures has been effective in encouraging disease managers (e.g. farmers and hunters) to adopt positive disease management practices?

- Economic (incentives, penalties)
- Social (Peer pressure, sense of collective action)
- Cultural (appealing to the 'good' farmer)

Can you point to any examples of where an **intervention** (e.g. campaign, voluntary initiative etc) has been effective in encouraging disease managers (e.g. farmers and hunters) to adopt positive disease management practices?

- What led to its success?

Conversely, are there any examples of where policies or interventions have not been effective?

- What led to its failure?

3. Stakeholders – approx 20 minutes [Use Blank Sheet of Paper with "TB" at the centre – Appendix 1]

Who are the people who have a 'stake' in controlling the disease? This could include organisations or individuals.

Who has the most influence in controlling the disease?

Who has least influence?

Are there any connections between these people/organisations that:

- Contribute towards more effective TB control?
- Limit the progress towards TB control?

Can you give any examples of where these individuals/organisations/relationships have contributed to the adoption of policies and/or 'best practice' in disease management on farms?

- E.g. biosecurity measures
- Trading decisions
- Others

4. Communications – approx 20 minutes

How are new policies and developments in 'best practice' for TB control communicated to disease managers?

[Ask the interviewee to trace on a piece of paper the chain of information from issuer via linkages to on-farm practice – first ask what the 'ideal' version should be and then ask what actually happens, picking up on the disconnects and why they occur]

What are the obstacles that government/advisors face in communicating information on TB?

How does this affect disease control on farm?

How might this be improved?

Can you give any examples of cases where best practice communication has been effective in producing positive changes in disease management practices?

[note that 'communication' can take different forms – written, verbal, via different intermediaries etc]

5. Michigan – Minnesota – approx 20 minutes

Why has Minnesota been more effective in their attempts to control TB?

- Control measures applied

- Economic influences

- Social influences

What are the main 'lessons learnt' from both the Michigan/Minnesota experience?

6. Disease control at the Farm level – approx 10 minutes

Are there any key documents that you suggest I look at?

- Overarching policy documents
- Overviews of activities/policies aimed at encouraging disease managers to adopt positive disease management practices
  - TB
  - Other endemic diseases e.g. Johnes, BVD

Are there any key people or organisations that I should be speaking with?

Are there any questions that I should be asking that I may have missed?

Appendix 1: TB Stakeholders

bTB

Stakeholders = people/organisations who can influence the reduction of disease
Influence: Red = negative Green = Positive
